# Genetic variation in *GABRA2* moderates peer influence on externalizing behavior in adolescents

**DOI:** 10.1002/brb3.291

**Published:** 2014-10-13

**Authors:** Sandra Villafuerte, Elisa M Trucco, Mary M Heitzeg, Margit Burmeister, Robert A Zucker

**Affiliations:** 1 Department of Psychiatry, University of MichiganAnn Arbor, Michigan; 2 Molecular & Behavioral Neuroscience Institute, University of MichiganAnn Arbor, Michigan; 3 Addiction Research Center, University of MichiganAnn Arbor, Michigan; 4 Department of Human Genetics, University of MichiganAnn Arbor, Michigan; 5 Department of Computational Medicine and Bioinformatics, University of MichiganAnn Arbor, Michigan

**Keywords:** Adolescence, externalizing, *GABRA2*, gene–environment interaction, peer delinquency

## Abstract

**Background:**

Genetic predisposition and environmental influences are both important factors in the development of problematic behavior leading to substance use in adolescence. Involvement with delinquent peers also strongly predicts adolescent externalizing behavior. Several lines of evidence support a role of *GABRA2* on externalizing behavior related to disinhibition. However, whether this genetic association is influenced by the environment such as peer behavior remains unknown.

**Methods:**

We examined the moderating role of *GABRA2* genetic variation on the socialization model of delinquent peer affiliation (at ages 12–14 years) on externalizing behavior (at ages 15–17 years) in the Michigan Longitudinal Study (MLS) adolescent sample.

The sample consisted of 244 adolescents (75 females and 152 with at least one parent with a *DSM-IV* lifetime alcohol dependence/abuse diagnosis). Peer delinquent activity reported by the participant and teacher-reported adolescent externalizing behavior (Teacher Report Form (TRF) were assessed.

**Results:**

No main effect of the *GABRA2* SNP rs279826, which tags a large haplotype, on externalizing behavior was observed. However, there was a statistically reliable *GABRA2* × peer delinquency interaction. The effect of peer delinquent involvement on externalizing scores and the rule breaking subscale is significantly stronger for those with the GG genotype compared to A-carriers, whereas there was no effect of genotype on externalizing in the absence of peer delinquent involvement. No interaction was observed for the aggression subscale.

**Conclusion:**

Our results suggest that the genetic effect of *GABRA2* on externalizing behavior, more specifically on rule breaking is, at least in part, due to its effect on susceptibility to environmental exposure (i.e., peer delinquency).

## Introduction

Genetic predisposition and environmental influences are both important factors in the development of problematic behavior (e.g., externalizing, substance use) (Rhee et al. 2003). Since the risk originates in adolescence, there may be multiplicative influences of genes and social environment unique to this developmental stage. Externalizing behaviors in adolescence such as aggression and rule breaking (e.g., defiance, theft, and vandalism) are actions that harm others. While aggression decreases, rule breaking increases during adolescence from early through mid-to-late adolescence (Bongers et al. 2004; Pepler et al. 2010). These traits are genetically influenced (Hicks et al. 2004). One salient environmental predictor of problem behavior and subsequent substance use at this developmental stage is the influence of delinquent peers (Rubin et al. 2006) which appears to be more pronounced during early adolescence (before 15-year-old) (Kendler et al. 2011) and have a strong influence on the trajectory of externalizing behaviors (Steinberg et al. 1994; Kendler et al. 2008). A twin study in adolescence suggests that genetic influences on delinquent behavior increase with age (Burt and Neiderhiser 2009).

Two processes have been proposed to account for strong associations between peer and adolescent behavior: selection and socialization. Selection models propose that delinquent involvement and substance use lead to friendships with peers who support delinquency and substance use, while peer socialization models suggest that certain friendships lead to delinquency and substance use. Research generally supports both selection and socialization (Mercken et al. 2009), but socialization is more prominent in the early stages of alcohol and drug use (Wills and Cleary 1999). Longitudinal studies support the evidence of socialization effects from peer drug use to adolescent substance use (Wills and Cleary 1999).

Research conducted on the involvement of *GABRA2* in alcohol use, impulse control behavior, and environment is indicative of its role in behavior characterized by hyperexcitability, impulsivity, or externalizing spectrum disorder (Dick et al. 2006b, 2009; Villafuerte et al. 2012). Those high in the externalizing trait tend to be more impulsive and sensation seekers (Rubin et al. 2006). Furthermore, genetic variations in this gene have predicted differences in response to the environment. To illustrate the complexity of these relations, Dick et al. (2006a) reported both gene–environment correlation (rGE) and Gene × Environment interaction (G x E) effects of *GABRA2* with marital status and with alcohol dependence. A *GABRA2 *× Parental monitoring interaction has also been shown with externalizing behavior in adolescence; high levels of parental monitoring diminished the association of *GABRA2* variants on trajectories of externalizing behavior (Dick et al. 2009).

The mechanisms by which *GABRA2* may influence these behaviors are starting to emerge. The nucleus accumbens (NAcc) in the mesolimbic dopamine system is important for its integrative role of directing behavior from the limbic system and prefrontal cortex (Grace et al. 2007). The GABA system exerts its inhibitory regulation of dopaminergic function in the NAcc (Steffensen et al. 1998). Increased NAcc activation has been associated with behavioral traits such as impulsiveness (Forbes et al. 2009; Hahn et al. 2009), sensation seeking (Bjork et al. 2008), and externalizing behaviors (Yau et al. 2012). In adolescents, genetic variation in *GABRA2* is associated with individual differences in NAcc activation during incentive anticipation, an aspect related to dopamine-specific motivated behaviors (Heitzeg et al. 2014).

Given the clear influence of delinquent peers upon externalizing behavior (i.e., peer socialization model), and the evidence for the association of *GABRA2* with increased susceptibility to social contexts, we prospectively investigated the moderating role of *GABRA2* genetic variation on the influence of peer delinquency on externalizing behavior among adolescents from the Michigan Longitudinal Study (MLS), a sample enriched with adolescents at risk to develop problem behavior, an important feature to detect small genetic effects. Specifically, we tested whether *GABRA2* risk variants increase the degree of susceptibility to delinquent peers on externalizing behavior and its subscales, rule breaking and aggression.

## Material and Methods

### Subjects and assessment

The sample consisted of 244 adolescents (75 females and 152 with at least one parent with a *DSM-IV* lifetime alcohol dependence/abuse diagnosis) from the MLS. This is an ongoing multiwave, community-recruited, prospective study of families of men with a drunk-driving conviction and AUD diagnosis who were living with a 3- to 5-year-old son/daughter and the biological mother at time of recruitment (father mean age 32; range 22–46 at baseline). Mother's diagnosis was free to vary. The study began recruitment in 1985. In addition, control families of like family composition but without a history of substance abuse were recruited from the same or socioeconomically comparable neighborhoods. Families identified during the community canvass for controls that also had a male parent with AUD diagnosis were recruited as well (Zucker et al. 1996). For this study, adolescents came from 175 families; 113 did not have any other siblings (64.6%) in the study, 55 (31.4%) had one, and 7 (4.0%) had two siblings in the study. The great majority was of European American descent (232; 95%) with 6 (2.5%) African American or biracial and 6 (2.5%) White Hispanic ancestry. Behavioral and alcohol or drug measures, appropriate for age, were extensively assessed on all family members at 3-year intervals. Written informed consent was obtained from the parents of all participants after the nature of the study had been explained to them; assent was also obtained from the offspring. The protocol was approved by the Institutional Review Board at the University of Michigan.

### Externalizing behavior

Teacher report of adolescent externalizing behavior and the subscales of rule breaking/delinquency and aggression at ages 15–17 years were assessed using *t*-scores from the Teacher Report Form (TRF) developed by Achenbach (1991). Teacher report was selected because it reflects behavior in the environment where peer interactions often take place and it is not confounded with the environmental measure (involvement with delinquent peers) reported by the subject. Moreover, the TRF has been demonstrated to be the best predictor of referral for mental health services compared to mother-, father-, or self-reports (Stanger and Lewis 1993). The test–retest reliability of the TRF for problem scores was found to be high (0.92) over a mean interval of 15 days. Stability was good over a 2- or 4-month period. Interrater agreement was similar for teachers seeing pupils under different conditions (*r *= 0.54 for problem scores).

### Peer delinquency

Involvement with delinquent peers was measured as part of the Peer Behavior Profile (Hirschi 1969) that assesses aspects of the participant's friendship network. It asks the participant to report on how many of their peers are involved in specific activities, and the activities listed cover a wide range of behavior. Responses to an eight-item subscale ask the participant to report what proportion (based on a five-point scale ranging from “almost none” to “nearly all”) of his or her friends are involved in delinquent activity such as stealing, shoplifting, vandalizing private property, staying out all night without permission, run away from home, have been in juvenile court, detained by police, or spent time in jail. The score is the mean value of the eight items for participants aged 12–14 years. The internal consistency for this sample was adequate (Cronbach's *α* coefficient 0.87).

### Genotyping

Genotype data were available for three SNPs (rs279826, rs279858, and rs279827) in strong linkage disequilibrium that capture the information of a long haplotype block of 109 kb where previous associations have been reported with alcohol dependence and impulsive-related traits. We report results for SNP rs279826 (intron 4) for which we have more genotyping data and similar results were found with the other two SNPs. Testing haplotypes produced similar results (data not shown). Details of the genotyping method are reported elsewhere (Villafuerte et al. 2012). Genotype frequencies for rs279826 are in Hardy–Weinberg equilibrium: AA 27.4% (*N *=* *67), AG 48.8% (*N *=* *119), and GG 23.8% (*N *=* *58).

### Data analytic plan

Normality distributions of peer delinquency and teacher-reported externalizing behaviors and subscales of rule breaking and aggression were assessed. Only one of the three variables (peer delinquency) was not normally distributed; therefore, a log transformation was performed (skewness = 1.74 and kurtosis = 3.88 for transformed variable).

It is important to note that the sample included siblings from the same nuclear family. Adolescents within a given family cannot be treated as independent observations since siblings are nested within families. Adolescents within one family are likely to share characteristics (e.g., family SES, socialization, genotypes, etc.) and be more similar to each other compared to adolescents from a different family (Raudenbush and Bryk 2001; Jenkins et al. 2003). That is, it is likely that there is less variation between children from the same family in their delinquent behaviors than between adolescents from different families. Additionally, siblings share about 50% of their DNA sequence. Ignoring the nested multilevel structure of the data can bias results and lead to incorrect inferences given attenuated standard errors (Raudenbush and Bryk 2001).

Therefore, an intercept only model was estimated (random intercept with no predictors) using the mixed procedure in SAS version 9.3 (SAS Institute Inc., 2011). Interclass correlations (ICCs) (Raudenbush and Bryk 2001) were calculated from clustering effects of family for all study outcomes (teacher-reported externalizing behaviors and the subscales rule breaking and aggression). Results suggested that the amount of variance accounted for by the clustering effect of families on study outcomes was significant for rule breaking (ICC = 0.24, *P* < 0.001) but not significant for externalizing (ICC = 0.11, *P* = 0.08) or aggression (ICC = 0.11, *P* = 0.07). Accordingly, a random coefficients (random intercept with adolescent-level predictors) hierarchical linear model accounting for family clustering was estimated across models to have consistency. Adolescent-level or level-1 predictors included the following: sex, race, *GABRA2,* peer delinquency, and the interaction term. Moreover, correlated residuals were accounted for using the REPEATED statement.

Externalizing and the subscales of rule breaking and aggression at age 15–17 years old were analyzed in separate models and *GABRA2* SNP rs279826 was included as a predictor. Adolescent biological sex and race were also included as control variables. Of interest was one-two-way interaction (*GABRA2 *× peer delinquency) to assess how peer delinquency may interact with genetic susceptibilities to predict externalizing behavior, aggression, and rule breaking 3 years later. To eliminate nonessential multicollinearity, first-order terms (i.e., covariates and peer delinquency) were standardized. *GABRA2* SNP rs279826 was dummy coded (0 = A-carriers, 1 = GG) prior to forming the cross-product interaction term as recommended (Aiken and West 1991).

An advantage of the prospective design of this study was that it established temporal precedence between peer delinquency, and the outcomes of interest assessed 3 years later. Cohen and Cohen (1983) recommended guideline of using values corresponding to one standard deviation (SD) above and below the sample mean for peer delinquency was used to probe the interaction. Given that the value corresponding to one standard deviation below the mean was outside the range of possible responses, a value of −0.9 was used instead. Finally, an online computational tool was used to probe significant interactions as well as providing a formal analysis of the regions of significance (Preacher et al. 2006). This provides a significance test of the association between the moderator and the outcome at all values of the independent variable that fall within the range of interest.

## Results

### *GABRA2* and peer delinquency interaction on externalizing behavior

**Figure 1:**
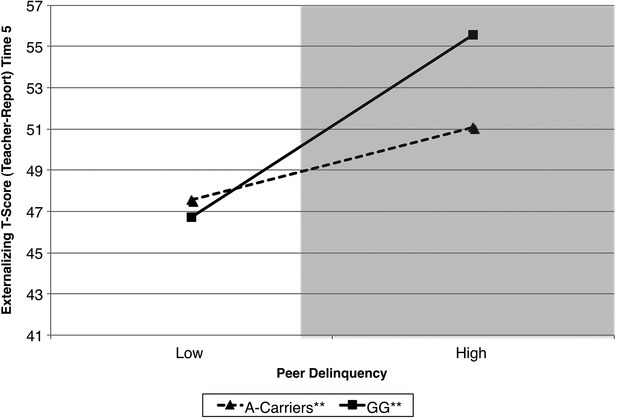
Peer delinquency and externalizing t-scores by genotype. The shaded area represents the region of significance.

Results predicting adolescent externalizing behaviors are presented in Table 1. The level-1 predictors in the model accounted for approximately 22% of the variance in externalizing *t*-scores. The first-order effects suggested that high levels of peer delinquency were prospectively associated with increases in externalizing behavior 3 years later. There was no evidence for first-order effects of covariates or *GABRA2* on externalizing *t*-scores. There was evidence for a statistically reliable *GABRA2 *× Peer delinquency interaction term. As depicted in Figure1, the simple slope of peer delinquency on externalizing behavior was statistically significant for both A-carriers (β = 1.85, *P *<* *0.001) and adolescents with the GG genotype (β = 4.65, *P *<* *0.001) although this association was stronger for adolescents with the GG genotype. At low levels of peer delinquency, adolescents with the risk genotype (GG) had lower rates of externalizing behavior compared to A-carriers. In contrast, at high levels of peer delinquency, adolescents with the risk genotype, GG, had greater rates of externalizing behavior compared to A-carriers. The lower and upper bounds of regions of significance were −9.60 and −0.25, respectively. This indicates that these two regression lines were significantly different for all possible points when the score of peer delinquency was lower than −9.60 (outside of the range of responding) or higher than −0.25 (represented by the shaded area in Fig.1). This suggests that it is only at average to high levels of peer delinquency that the regression lines for A-carriers and those with the GG genotype differ significantly in terms of externalizing behavior.

**Table 1:** Multilevel linear regression model for teacher-reported externalizing, rule breaking and aggression 3 years later

	Coefficient	SE	*t* value
Model predicting externalizing			
Intercept	49.22***	0.67	73.78
Sex	−0.34	0.50	−0.69
Race	1.20	0.88	1.36
*GABRA2*	1.68	1.24	1.35
Peer delinquency	1.85**	0.60	3.08
*GABRA2 *× Peer delinquency	2.80*	1.30	2.16
Model predicting rule breaking			
Intercept	53.75***	0.46	116.61
Sex	−0.45	0.34	−1.32
Race	1.59**	0.61	2.63
*GABRA2*	0.76	0.86	0.89
Peer delinquency	1.27**	0.41	3.09
Gene × Peer delinquency	2.58**	0.89	2.90
Model predicting aggression			
Intercept	53.11***	0.46	116.37
Sex	−0.37	0.33	−1.13
Race	0.83	0.60	1.39
*GABRA2*	1.39	0.84	1.65
Peer delinquency	1.07**	0.40	2.64
*GABRA2 *× Peer delinquency	1.25	0.87	1.44

* *P *< 0.05,

** *P *< 0.01,

*** *P *< 0.001.

### *GABRA2* and peer delinquency interaction on rule breaking and aggression

**Figure 2:**
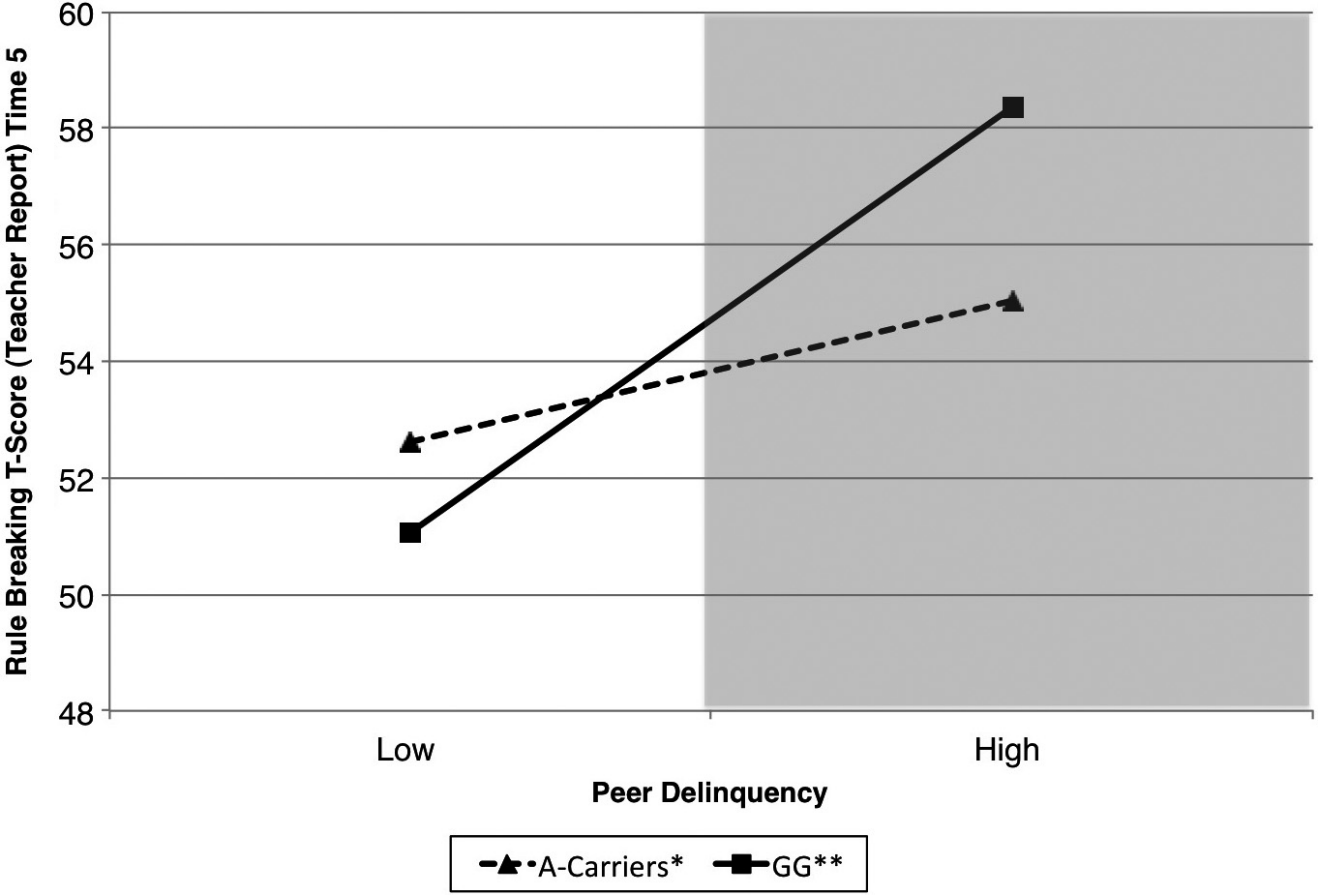
Peer delinquency and rule breaking t-scores by genotype. The shaded area represents the region of significance.

Rule breaking, a major component of externalizing behavior, was found to be significantly affected by race, peer delinquency, and interaction with *GABRA2* (Table 1). The level-1 predictors in the model accounted for approximately 13% of the variance in rule breaking *t*-scores. The first-order effects suggested that race was associated with rule breaking behavior. Namely, non-European American (i.e., African American and Hispanics) adolescents had higher rates of rule breaking behavior compared to European American adolescents. In addition, first-order effects suggested that high levels of peer delinquency were prospectively associated with higher levels of rule breaking behavior 3 years later. No main effects of *GABRA2* were observed. However, as hypothesized, there was evidence for a statistically reliable *GABRA2* × Peer delinquency interaction. As depicted in Figure2, the simple slope of peer delinquency was statistically significant for both A-carriers (β = 1.27, *P *<* *0.01) and adolescents with the GG genotype (β = 3.85, *P *<* *0.001) although this association was stronger for adolescents with the GG genotype as hypothesized. This suggests that at low levels of peer delinquency, adolescents with the risk genotype (GG) have lower rates of rule breaking behavior compared to A-carriers. In contrast, at high levels of peer delinquency, adolescents with the risk genotype have greater rates of rule breaking behavior compared to A-carriers. Yet, the lower and upper bounds of regions of significance were −2.07 and −0.13, respectively. This indicates that these two regression lines were significantly different for all possible points when the score of peer delinquency was lower than −2.07 (outside of the range of responding) or higher than −0.13 (represented by the shaded area in Fig.2). Similar to the previous model, this suggests that it is only at average to high levels of peer delinquency that the regression lines for A-carriers and those with the GG genotype differ significantly in terms of rule breaking behavior.

The other subcomponent of externalizing, aggression was significantly affected by peer delinquency such that affiliation with delinquent peers predicts higher levels of aggression 3 years later. No other predictors were significant for this outcome (Table 1). Moreover, there was no evidence for a *GABRA2 *× Peer Delinquency interaction on aggressive behavior.

## Discussion

The present study provides further evidence on the contribution of *GABRA2* genetic variation on the strength of the relation between deviant peer affiliation and externalizing behavior. Furthermore, our results suggest that the influence of *GABRA2* genetic variation is on one specific subcomponent of this behavior, namely rule breaking but not aggression. Consistent with study hypotheses, peer delinquency positively predicts externalizing behavior. When externalizing behavior was analyzed separately by its subcomponent parts, there was evidence for a significant effect of *GABRA2* as a moderator on rule breaking behaviors, but not on aggression. The association was stronger for adolescents carrying the GG genotype compared to A-carriers. This suggests that adolescents with the GG genotype may be more susceptible to the influence of delinquent peer involvement on externalizing behavior driven by rule breaking but not aggression. Moreover, the regions of significance analysis suggest that the interaction is most consistent with a diathesis-stress model (Zuckermann 1999), which suggests that some individuals are more vulnerable to adverse effects of environmental stressors (e.g., delinquent peers) due to their genetic composition. That is, adolescents with the GG genotype do not differ from A-carriers at low levels of peer delinquent; it is only when exposed to adverse social contexts (i.e., higher levels of contact with delinquent peers) that differences emerge. This is in contrast to the differential susceptibility model (Belsky et al. 2009), which suggests that adolescents with genotypes traditionally conferring risk may actually be more affected by adverse as well as adaptive environments. Failure to detect significant differences between GG and A-carriers in the low peer delinquent scenario may be explained by the fact that low delinquent peer contact does not represent involvement with a supportive environment (e.g., peers involved in academics or church). Furthermore, the lower bound of region of significance was outside the range of responding. Another reason may be that the sample is overrepresented by subjects associating with peers with high levels of delinquency, thus with a higher power to detect significant interactions in the upper bound. We cannot rule out the possibility that this same genotype (i.e., GG) may confer lower levels of externalizing in a more supportive peer environment compared to A-carriers, consistent with the differential susceptibility theory (Belsky et al. 2009). Indeed, when probing interactions in this sample with measures of positive peer involvement, a pattern consistent with differential susceptibility emerged (Trucco et al. 2014). In summary, we have demonstrated that the influence of peer delinquency upon externalizing behavior was moderated by genetic variation in *GABRA2* that in turn may impact risk for substance use. Risk factors in alcohol initiation in adolescents include both externalizing behavior and affiliation with deviant peers (Donovan 2004). We also ruled out the possibility that genetic predisposition, in the form of the *GABRA2* SNP, influenced the choice of peers by testing gene–environment correlation (rGE).

Our results are consistent with twin studies that suggest that in adolescence the strong effect of the environment on externalizing is modulated by genetic effects (Kendler et al. 2011). Furthermore, these results are largely consistent with the literature supporting the role of peer influence as a predictor of adolescent externalizing behavior. These studies have shown that increased association with deviant peers at ages 12–14 years predict more serious forms of externalizing behavior (e.g., covert forms of antisocial behavior) (Granic and Patterson 2006) and socialization effects from peer drug use to adolescent substance use (Wills and Cleary 1999). Here, we prospectively examined the socialization effects in the interplay between peer delinquency and *GABRA2* during adolescence on externalizing behavior, rule breaking, and aggression 3 years later (i.e., 15–17 years old). As expected, the association with deviant peers in early adolescence predicts externalizing and rule breaking behavior 3 years later. However, there was no main effect of *GABRA2* SNPs, highlighting the importance of the social environment in order for the moderating effect of *GABRA2* to emerge in this developmental period. Nevertheless, we could not rule out the direct effect of *GABRA2* on these dimensions since later in adulthood the genetic predisposition emerges in more stable measures of behavior like impulsiveness (Villafuerte et al. 2012, 2013).

This study contributes to the increasing literature on the effect of *GABRA2* on behaviors characterized by poor effortful control, namely externalizing behavior in adolescence and impulsive personality in adults, both risk factors for alcohol use disorder (AUD). However, the direction of the association is not clear as evidenced by prior work. Several authors have reported the minor allele of the SNPs in high LD as the risk allele in AUD (Covault et al. 2004; Edenberg et al. 2004; Lappalainen et al. 2005; Pierucci-Lagha et al. 2005; Enoch et al. 2006; Fehr et al. 2006; Bauer et al. 2007) and impulsiveness (Villafuerte et al. 2012) while other authors have reported the major allele as the risk allele for AUD (Agrawal et al. 2006; Lind et al. 2008; Soyka et al. 2008), conduct disorder (Dick et al. 2006b), and externalizing disorder (Dick et al. 2009). The present evidence strongly suggests that the minor *GABRA2* allele plays a role in behavioral disinhibition processes. Nonetheless, independent replications are still needed to determine whether the diathesis-stress model will be replicated.

Interestingly, the non-European American adolescents (African American or Hispanics) in our sample exhibited higher scores of rule breaking (but not externalizing) than those from European American ancestry. We consider this result with caution since only 5% of participants were from these minority groups. Nevertheless, in other work in children followed through adolescence African American participants exhibited higher baseline impulsivity scores than European Americans (Pedersen et al. 2012).

### Strengths and limitations of the present study

A unique aspect of the sample in this study is that it comprised adolescents with both high (62%) and low risk to develop AUD (based on parental diagnosis). Thus, the variability in behavior and environmental exposure in this sample may facilitate the detection of these nuanced genetic influences. However, our results cannot be generalized to other populations with other type of risk (e.g., drug use) or no risk. Another important characteristic is the prospective nature of the study, where the gene–environment interaction predicted future externalizing behavior, thus establishing a temporal relation of the gene–environment interaction effect upon outcome.

The genetic effect size in these complex scenarios is generally very small requiring adequate sample size. We were not able to detect any significant genetic main effect on externalizing behavior and the subscales, rule breaking and aggression. This may be due to the small sample size or the lack of a main effect at the age of 15–17, or most likely, to the strong effect that involvement with delinquent peers has upon these relationships. Finally, replication of these results is needed to further understand the role of GABRA2 on the relation of social context and behavior in adolescents.
